# Excision of the Tongue

**Published:** 1881-08

**Authors:** 


### EXCISION OF THE TONGUE.

Mr. William Stokes, of Dublin, at a recent meeting of the Clinical Society of London (*Lancet*, April 30, 1881), read a paper on this subject. He commenced by alluding to the change that surgical opinion has lately undergone in reference to the merits of excision of the tongue, and mentioned the views of Professor Gross and Mr. Collins on this subject. The particulars of six cases in which the writer had excised the tongue were then briefly stated, in which relief from suffering caused by enlargement of the tongue, difficulty of deglutition and articulation, discharge and pain, was obtained in all as an immediate result of the operation. In two of the cases a considerable time—twenty-two months and eighteen months—elapsed without any recurrence of the disease; in two others three months and four months elapsed; in one the patient was lost sight of immediately after the wound healed, and in only one of the cases was almost immediate return of the disease observable. Performing the operation at an early period of the development of the disease, if possible before glandular complication, was strongly advocated, and Sir J. Paget's views stated, as regards the small risk attending the operation.

The question of unilateral or bilateral ablation was then discussed, and the advantages of the latter pointed out. The question of what is the safest and best method of excision was then considered, as well as the two special dangers of the operation, hemorrhage and septic complications. The author observed that removal of the tongue by a cutting operation is not only more liable to be followed by hemorrhage, but also by septic infection, the outcome of which latter is in a large number of cases either pulmonary gangrene or septic pneumonia. These are not observable at all to the same extent in the cases operated on by the ecraseur. In proof of this, the statistics of Dr.

Scheffer and Mr. Collis were considered, in the former of which it was found that the percentage of mortality from septic causes in the cases operated on by incision reached the startling figures of 60, and in those of Mr. Collis 61. Mr. Barker's cases were also alluded to, but the number of cases (3) operated on by incision, in which there were no septic consequences, was not considered sufficiently large to materially affect the conclusions arrived at.

The disadvantages of certain of the ecraseur operations were then pointed out, and the author concluded by describing the method of operation he preferred. He commences by transfixing the cheek at a point corresponding to the last molar of the lower jaw, and makes an incision downwards and forwards towards the angle of the mouth, terminating a few lines above it. All bleeding vessels being secured, and the parts retracted, a ligature is passed through the tip of the tongue to facilitate the drawing forwards of the organ by an assistant. This being done, a straight Liston's needle, armed with a double strand of carbolized silk, is passed through the base of the tongue at a point behind the foramen cæcum. In front of this the chain of an ecraseur is passed round the tongue, and the organ gradually severed. This portion of the operation is done very slowly, taking from thirty-five to forty-five minutes. The author never witnessed in any of his cases any secondary hemorrhage, immediate or remote. The wound in the cheek is brought together by a few points of interrupted suture. The free ends of the strand of silk passed through the base of the tongue are then fastened temporarily, by adhesive plaster, to the side of the cheek or forehead. This double thread, which, in the event of secondary hemorrhage or traction, would be of much assistance in enabling the surgeon to draw forwards the base of the tongue and secure the bleeding vessels, is removed, usually on the third or fourth day after the operation. The author believed that, by the use of the ecraseur, employed in the manner he described, coupled with antiseptic measures during the healing of the wound, the operation is attended with little pain, with the minimum of risk as regards either primary or secondary hemorrhage, or those septic troubles which, after all cutting operations, are so fruitful a means of raising the mortality attending this important operation.

Mr. Heath said that in this country the tongue was more freely operated upon than was assumed by the author. As to the question of growth after removal, in Mr. Barwell's case there was a considerable increase; and in his own case, where more than half was removed, the stump of the tongue nine years after appeared to have enlarged. The mode of operation was different in different cases. He had now entirely given up the galvanic ecraseur, in favor of the chain ecraseur, and had never found occasion either to pierce or incise the cheek; for by bending the ecraseur and pulling forward the tongue, the instrument could be applied far back. Whether a unilateral or bilateral operation should be performed depended on the merits of the case; and he agreed with Mr. Baker that a unilateral operation should be done when necessary; it also enabled the surgeon to examine the two halves of the organ when split in the median line.

Mr. Morrant Baker advocated the unilateral method when the disease was sufficiently limited. The median incision avoided the severance of arteries and rendered the detachment of the organ more easy, and it allowed of removal of the other half if the disease were found to encroach near the median line. He thought division of the cheek might be more often done with advantage, especially when the disease extended far back. It allowed also of a better control of hemorrhage. He thought the tongue could not grow after incision, but that an apparent increase in size might result from the contraction of the cicatrix. And as to the retention of speech, it must be remembered that the tongue was not wholly removed when only the portion in front of the foramen cæcum was excised. When completely removed, the mouth has a very different appearance; there is a large hollow in the place of the root of the tongue, and the arches of the palate are approximated. Mr. Gant had recently exhibited a case of nearly entire removal, which had been effected much on the plan adopted by Collis. That patient could hardly articulate.

Mr. Baker had not himself found tracheotomy necessary; but in a case where he assisted, tracheotomy had to be done on account of blood flowing into the trachea. A fresh hemorrhage occurred just before the removal of the tongue was completed, and the blood flowed more readily into the trachea than when there was no tube in it. Of course it might be said that the pharynx should have been plugged.

Mr. MacCormac pointed out that the trachea itself might have been plugged. He asked Mr. Stokes what measures had been taken to remove the diseased glands, which occurred in some of his cases. Permanent success depended upon this. Any modification of the operation, by splitting the cheek, etc., was justifiable if it facilitated the removal of the diseased tissues in the floor of the mouth. The difficulty of removal of infiltrated glands was considerable, and he should like to know whether the plan followed by Mr. Stokes enabled him to effect their removal. He was surprised to learn that an operation producing a crushed and bruised surface (as did the ecraseur) is followed by less risk of septic processes than a simple cut wound. However, the fact remained. He had once or twice divided the tongue without any hemorrhage of moment occurring.

Mr. Barker said that the list he had furnished to Mr. Stokes somewhat altered the relative rate of mortality after different methods of operation. At University College Hospital, from from 1871 to 1879 inclusive, there had been 51 cases of lingual epithelioma, in 34 of which excision was performed. 11 of these cases died in consequence of the operation; 5 from septic pneumonia; 2 from septic bronchitis; 2 from septicæmia; 1 from pyæmia, and 1 from œdema of the glottis. A large proportion thus died from septic causes. Of these cases 25 were operated on by the galvanic ecraseur, with 9 deaths; 7 by the ecraseur, and 1 death (from pyæmia); 3 by the knife, no deaths; and 1 by ligature, fatal (from septicæmia.) These figures, although few, condemn the galvanic ecraseur. Did Mr. Stokes include the galvanic ecraseur in his statistics? Mr. Barker agreed with Mr. MacCormac as to the preference to be given to the knife. The tongue should be split in the median line, and the base cut across, this measure allowing of the ready control of bleeding vessels and of their ligature in the ordinary way, and was far less open to danger of septic trouble than after crushing. Tracheotomy was sometimes called for in cases where much hemorrhage was to be anticipated, or the diseased tissues were of wide extent. The risk from hemorrhage could then be met by plugging the trachea, or by means of a sponge in the pharynx. In three cases he had no trouble in retaining a sponge there; but if the tracher were to be plugged, Trendelenberg's tampon should be used, care being taken in its inflation, since, as Dr. Semon had shown, its over-inflation caused dyspnœa.

Dr. Douglas Powell asked Mr. Barker what was meant by "septic" bronchitis and "septic" penumonia. Did he imply that in such cases there was general septic infection; and were other organs affected, as in such septicæmia? If there were only bronchitis or pheumonia, that might possibly be produced by the passage of irritant matter into the bronchi or lung, and would be met by measures preventing such gravitation.

Mr. T. Smith asked whether Mr. Stokes made any incision in the sublingual tissues. He believed that only a portion of the tongue could be pulled forward, and that the organ could not be wholly removed unless the subjacent tissues were divided. As he understood it, the septic pneumonia of these cases is produced by blood, etc., trickling down the air-passages. The discharges were more copious after the use of the galvanic ecraseur, owing to the slough it produces.

Mr. Marsh, in two or three instances of unilateral excision, had been disappointed with the result, the longitudinal cicatrix holding the tongue down, and the muscles of the remaining half turning it over, a condition giving rise to discomfort. It was plain that opinion was adverse to the galvanic cautery. He was acquainted with secondary hemorrhage following its use, and with one or two of fatal complications. By first dividing the muscles attaching the tongue to the lower jaw the base of the epiglottis can be reached. The experience of St. Bartholomew's

Hospital did not accord with the statistics given by Mr. Barker.

Dr. Coupland urged Mr. Barker to reply to Dr. Powell's question. In post mortem examination of cases of cancer of the tongue, foci of broncho-pneumonia were common, and to be attributed, not to septic infection in the ordinary sense, but to the inhalation of irritant and putrid materials.

Mr. Barker said the question was an important one. No doubt sometimes there were pulmonary abscesses of truly pyæmic (embolic) kind; but the lungs might be also affected by blood trickling down the trachea during the operation, or fetid discharges similarly passing; also, he believed, by the simple inhalation of very acrid emanations from these wounds. In one instance the house-surgeon and nurse in attendance on the case became affected with sore throat. He did not think these different modes of lung complication should be confused.

Dr. Powell said the point was one of great practical importance. He had asked if there was any evidence of general blood poisoning to which the lung disease was secondary. If in all such cases the infection was local, then a remedy might be sought in antiseptic inhalations and in enforcing a prone position, so that the blood and discharges might not pass down the air-passages.

Mr. Barker said that in one case there was distinctly a general pyæmia, developing long after the operation, with abscesses in the liver and in the vicinity of the kidney, as well as the lung. In the other cases there was no involvement of other organs.

Mr. Stokes, in reply, said that he had not included Mr. Barker's statistics in his lists, but he had made a percentage mortality of about sixty in the cases of simple excision by the knife; and he admitted that it was remarkable that there should be apparently less danger from septicæmia in such cases than in those treated by the cautery. He was glad to find Mr. Marsh prefering the bilateral method, and could endorse what he had said as to the greater inconvenience both as regards deglutition and articulation resulting from one-half of the organ being excised than when both sides are removed. He knew of one case where the patient came to have the other half removed because of the difficulty in articulation and because it kept getting between his teeth. He was also pleased to hear Mr. Barker suggesting a preliminary division of the check, which greatly facilitates the operation. He had performed division of the sublingual tissues in such cases where the thickening and enlargement of the tongue called for it.

